# Infectivity in Skeletal Muscle of Cattle with Atypical Bovine Spongiform Encephalopathy

**DOI:** 10.1371/journal.pone.0031449

**Published:** 2012-02-21

**Authors:** Silvia Suardi, Chiara Vimercati, Cristina Casalone, Daniela Gelmetti, Cristiano Corona, Barbara Iulini, Maria Mazza, Guerino Lombardi, Fabio Moda, Margherita Ruggerone, Ilaria Campagnani, Elena Piccoli, Marcella Catania, Martin H. Groschup, Anne Balkema-Buschmann, Maria Caramelli, Salvatore Monaco, Gianluigi Zanusso, Fabrizio Tagliavini

**Affiliations:** 1 Instituto Di Ricoveroe Cura a Carattere Scientifico (IRCCS), Foundation “Carlo Besta” Neurological Institute, Milano, Italy; 2 Istituto Zooprofilattico Sperimentale del Piemonte, Liguria e Valle d'Aosta, Torino, Italy; 3 Istituto Zooprofilattico Sperimentale della Lombardia ed Emilia Romagna, Brescia, Italy; 4 Friedrich-Loeffler-Institut, Greifswald, Insel Riems, Germany; 5 Policlinico G.B. Rossi, University of Verona, Verona, Italy; Creighton University, United States of America

## Abstract

The amyloidotic form of bovine spongiform encephalopathy (BSE) termed BASE is caused by a prion strain whose biological properties differ from those of typical BSE, resulting in a clinically and pathologically distinct phenotype. Whether peripheral tissues of BASE-affected cattle contain infectivity is unknown. This is a critical issue since the BASE prion is readily transmissible to a variety of hosts including primates, suggesting that humans may be susceptible. We carried out bioassays in transgenic mice overexpressing bovine PrP (Tgbov XV) and found infectivity in a variety of skeletal muscles from cattle with natural and experimental BASE. Noteworthy, all BASE muscles used for inoculation transmitted disease, although the attack rate differed between experimental and natural cases (∼70% versus ∼10%, respectively). This difference was likely related to different prion titers, possibly due to different stages of disease in the two conditions, i.e. terminal stage in experimental BASE and pre-symptomatic stage in natural BASE. The neuropathological phenotype and PrP^res^ type were consistent in all affected mice and matched those of Tgbov XV mice infected with brain homogenate from natural BASE. The immunohistochemical analysis of skeletal muscles from cattle with natural and experimental BASE showed the presence of abnormal prion protein deposits within muscle fibers. Conversely, Tgbov XV mice challenged with lymphoid tissue and kidney from natural and experimental BASE did not develop disease. The novel information on the neuromuscular tropism of the BASE strain, efficiently overcoming species barriers, underlines the relevance of maintaining an active surveillance.

## Introduction

In 2004 an atypical form of bovine spongiform encephalopathy (BSE) termed BASE or BSE-L was identified in Italy through active surveillance [1] and subsequently recognized in different European countries, North America, Canada and Japan [1]–[6]. BASE affects relatively old cattle (all cases were older than 9 years) and differs from BSE with regard to the neuropathological phenotype, the biochemical profile of the disease-associated prion protein (PrP^Sc^) and the biological properties of the agent strain [1], [3], [7]. The neuropathological hallmark of BASE is the presence of PrP amyloid plaques and the preferential involvement of olfactory areas, hippocampus and thalamus with a relatively low involvement of the brainstem, as opposed to classical BSE. The molecular signature of BASE is a PrP^Sc^ type distinguished by a protease-resistant core (PrP^res^) of lower molecular mass than BSE-PrP^Sc^, with predominance of the monoglycosylated protein band by western immunoblot.

Intra-species transmission revealed that the clinical picture of BASE differs substantially from that of BSE, being characterized by mental dullness and amyotrophy rather than hyper-reactivity and aggressiveness [8], [9]. As a consequence, the recognition of BASE *in vivo* can be difficult and may represent a major challenge for passive surveillance. Transmission studies to transgenic mice overexpressing bovine PrP (Tgbov mice) showed that the BASE strain is more aggressive than the BSE strain [10]. Furthermore, Tg mice overexpressing human PrP as well as non-human primates are more susceptible to infection with BASE than with BSE [11]–[14]. Overall these data raise concern about the potential risk of transmission of BASE to humans and it is urgent to determine the presence and distribution of infectivity in peripheral tissues of BASE-affected cattle. To investigate this issue, we inoculated Tgbov mice with different peripheral tissues from experimentally and naturally BASE-affected cattle and found that various skeletal muscles contained infectivity and PrP-immunoreactive deposits within individual fibers.

## Results

### Skeletal muscle of cattle with experimental BASE contains infectivity

Transgenic mice overexpressing bovine PrP on a mouse PrP knock-out background (Tgbov XV line) [15] were injected both intracerebrally (i.c.) and intraperitoneally (i.p.) with 10% homogenate of *longissimus dorsi* muscle obtained from an Alpine brown cow intracerebrally infected with BASE and culled at terminal stage of disease (#995) [8] (Table 1). As positive control, groups of mice were inoculated with brain homogenates from the same experimentally BASE-infected cattle and from a natural BASE identified by active surveillance (#12966/07). No PrP^res^ was detected in muscle homogenate used for inoculation by immunoblot analysis without or with PrP^res^ enrichment with phosphotungstic acid (PTA) precipitation [16].

**Table 1:** Transmission of BASE to Tgbov XV mice following inoculation of different tissues.

Tissue sample	N° diseased/N° inoculated	Incubation time (days)	Survival time (days)
**EXPERIMENTAL BASE (#995)**			
Brain	5/5	186±10	215±9
*Longissimus dorsi* muscle	5/7	380±11	410±12
Kidney	0/9	/	>850
Spleen	0/7	/	>850
Lymph node	0/14	/	>850
**NATURAL BASE (#12966/07)**			
Brain	5/5	178±6	211±8
*Gluteus* muscle	1/7	370	396
*Intercostalis* muscle	1/9	498	541
Kidney	0/8	/	>850
Spleen	0/8	/	>850

Incubation and survival times are expressed in days as mean ± s.e.m.

All mice infected with experimental (5/5) or natural (5/5) BASE brain homogenate developed the disease with comparable incubation period (mean ± standard error of the mean [s.e.m.]: 186±10 and 178±6 days) and survival time (mean ± s.e.m.: 215±9 and 211±8 days). Noteworthy, five out of seven mice challenged with the *longissimus dorsi* muscle developed a progressive neurological disease with incubation periods ranging from 347 to 430 days (mean ± s.e.m.: 380±11 days) and survival times ranging from 364 to 430 days (mean ± s.e.m.: 410±12 days). As observed in mice infected with BASE brain tissue, clinical signs of disease consisted of lethargy, hindlimb weakness and amyotrophy, and weight loss.

**Figure 1:**
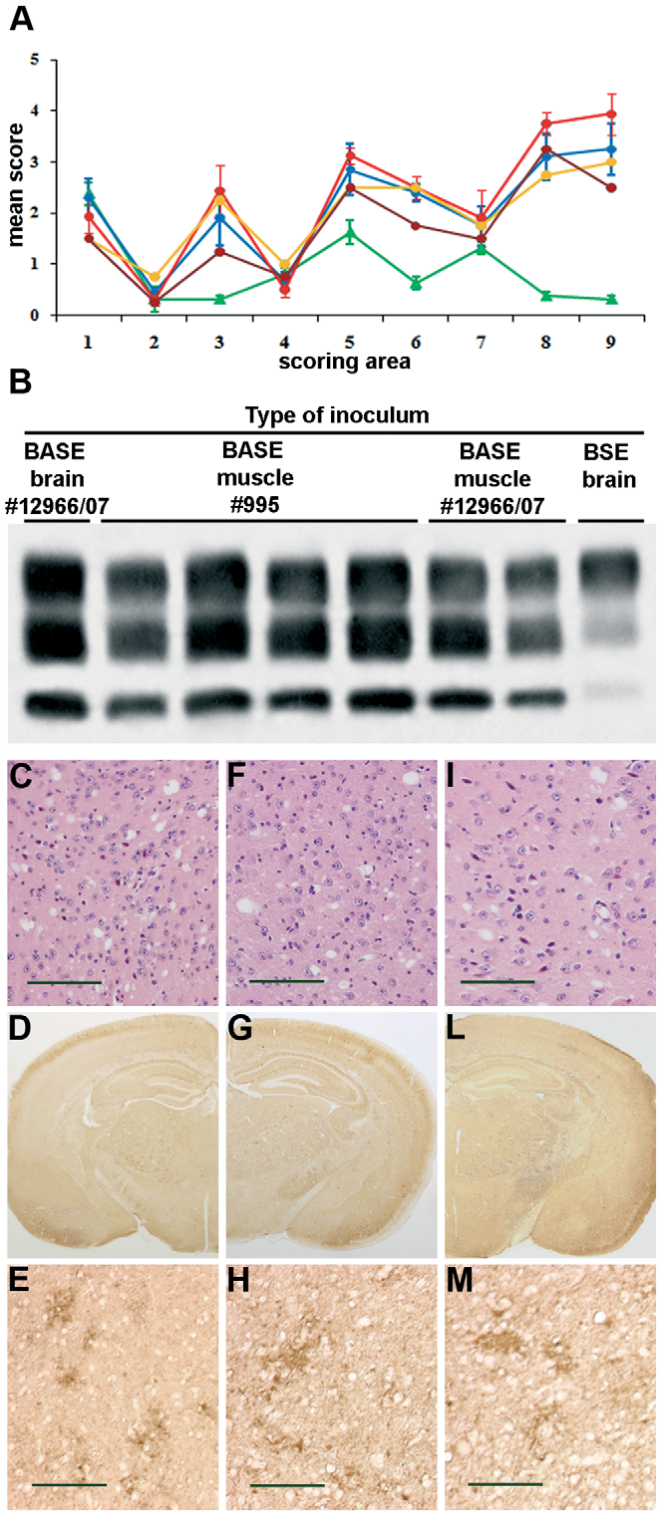
Transmission of BASE to Tgbov XV mice following inoculation of muscles from experimentally and naturally affected cattle. (A) Lesion profiles of mice infected with brain tissue from natural BASE (blue line) and BSE (green line), *longissimus dorsi* muscle from experimental BASE (red line) and *intercostalis* (bordeaux line) and *gluteus* (yellow line) muscles from natural BASE. Vacuolation was scored on a scale of 0–5 in the following brain areas: 1, dorsal medulla; 2, cerebellar cortex; 3, superior colliculus; 4, hypothalamus; 5, thalamus; 6, hippocampus; 7, septum; 8, retrosplenial and adjacent motor cortex; and 9, cingulated and adjacent motor cortex. Data are mean ± s.e.m. (B) Western blot analysis of proteinase K-digested brain samples of mice infected with brain homogenates from cattle with natural BASE (#12966/07) and BSE, *longissimus dorsi* muscle from cattle with experimental BASE (#995) and two different muscles from natural BASE (#12699/07). (C–M) Neuropathological changes of mice infected with brain (C–E) and muscle from cattle with experimental BASE (F–H), and muscle from cattle with natural BASE (I–M). Micrographs were obtained from corresponding areas of the thalamic region stained with haematoxylin-eosin (C,F,I) or labeled with the anti-PrP antibody 6H4. The severity of vacuolation and the type of PrP deposition, characterized by diffuse immunostaining of the neuropil with focal enhancement, is similar in all the samples analyzed. Scale bar = 100 µm.

The neuropathological profile, immunohistochemical pattern of PrP^res^ deposition and PrP^res^ type were identical in Tgbov XV mice challenged with BASE muscle or BASE brain, and differed from that of mice injected with the BSE strain. In fact, the histopathological lesion profile (i.e. extent of vacuolar degeneration in standard brain regions) was marked by major involvement of somatosensory cortex and superior colliculus as opposed to a substantial sparing of these regions in BSE-infected mice (Figure 1A). Immunohistochemistry showed diffuse PrP^res^ deposition in the cerebral cortex, particularly in the somatosensory region, basal ganglia, thalamus, hypothalamus, brainstem and cerebellum, accompanied by some focal PrP deposits, whereas amyloid plaques were absent as revealed by specific staining for amyloid (Figure 1C, D, E, F, G, H). The PrP^res^ type was characterized by prevalence of the monoglycosylated dominant profile and faster electrophoretic migration compared to BSE-infected mice (Figure 1B).

### Skeletal muscle of cattle with natural BASE contains infectivity

As a subsequent step in our study, we inoculated Tgbov XV mice both i.c. and i.p. with 10% homogenate of two different muscles (*intercostalis* and *gluteus* muscles) from a 14-year-old asymptomatic Piemontese cow with natural BASE (#12966/07) (Table 1). Transmission to mice was observed from both muscles, although the attack rate was less efficient than that of mice injected with the experimental BASE muscle. One out of seven mice challenged with intercostal muscle and one out of nine mice challenged with gluteal muscle developed clinical signs of disease with incubation time of 370 and 498 days, and survival time of 396 and 541 days, respectively. The lesion profile, the pattern of PrP^res^ deposition, the brain regional distribution and the biochemical type of PrP^res^ were identical to those observed in mice inoculated with muscle from cattle with experimental BASE (Figure 1A and Figure 1I, J, K, L, M).

### Skeletal muscle of BASE-affected cattle contains abnormal PrP deposits

**Figure 2:**
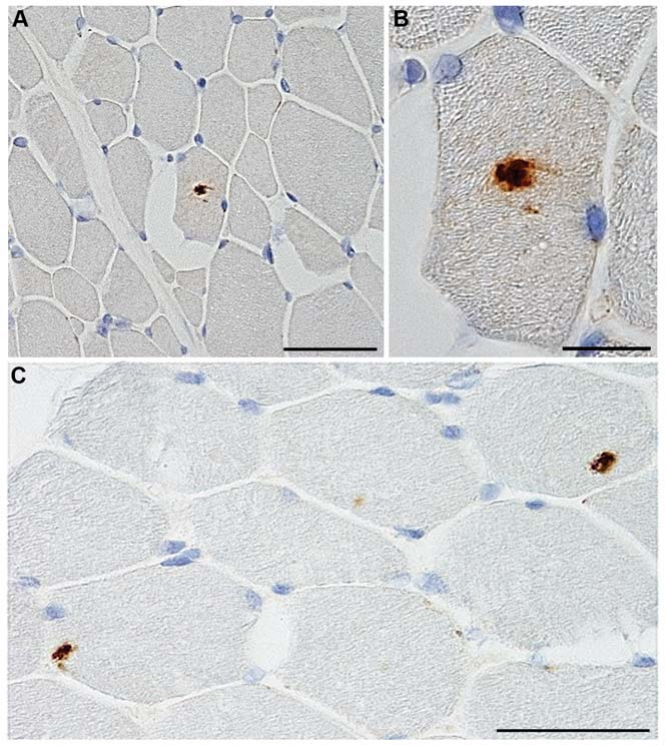
PrP deposition in the muscles of natural BASE cattle. (A–C) PrP deposits in *peroneus* muscle from a cattle with natural BASE (#126752/09). The PrP-imunoreactive material was found in isolated muscle fibers with a scattered distribution (A,C) and was localized inside the cytoplasm in the form of small amorphous aggregates or granular deposits (B). Scale bar = 100 µm for figure A and C;  = 20 µm for figure B.

Immunohistochemical analysis showed the presence of PrP deposits in 2 out of 6 muscles (i.e., *M. longissimus dorsi* and *M. pectoralis profundus*) obtained from the experimental BASE used for transmission studies (#995). Furthermore, immunoreactivity was detected in 4 out of 16 muscles (i.e., *M. trapezius*, *M. biceps femoris*, *M. semitendinosus* and *M. peroneus*) from a distinct case of natural BASE (#126752/09) (Tables 2 and 3). In both cases, PrP deposition was observed inside the cytoplasm of isolated fibers with a scattered distribution, mainly as small amorphous aggregates and, less frequently, in the form of granular deposits (Figure 2). PrP immunoreactivity was abolished by preabsorption of the antibody with a peptide comprising the antibody epitope. Muscle samples from negative control cattle consistently gave negative results. The PrP immunoreactive deposits did not show the staining properties of amyloid as revealed by absence of staining with the amyloid-binding dye X-34. Neither muscle atrophy nor inflammatory processes were observed in any of the muscle sections from natural BASE stained with hematoxylin-eosin, while muscles from experimentally infected cattle showed a remarkable amyotrophy [8]. WB analysis of samples of skeletal muscles used for immunohistochemistry did not reveal presence of PrP^res^ either without or with PTA precipitation.

**Table 2:** PrP immunohistochemistry of muscle samples from Alpine brown cow experimentally infected with BASE (#995).

	Skeletal muscle	PrP^res^ detection
**Forelimb**		
	*M. deltoideus*	−
**Thorax**		
	*M. pectoralis profundus*	**+**
**Rump**		
	*M. longissimus dorsi*	**+**
**Hindlimb**		
	*M. psoas*	−
	*M. gluteus*	−
	*M. gastrocnemius*	−

**Table 3:** PrP immunohistochemistry of muscle samples from the Holstein-Friesian cow affected by natural BASE (#126752/09).

	Skeletal muscle	PrP^res^ detection
**Neck**		
	*M. trapezius*	**+**
**Forelimb**		
	*M. triceps brachii*	−
	*Caput laterale mi. tricipitis brachii*	−
	*M. extensor carpi radialis*	−
	*M. extensor carpi ulnaris*	−
	*M. extensor digitorum lateralis*	−
**Thorax**		
	*M. pectoralis profundus*	−
	*M. intercostalis*	−
**Rump**		
	*M. longissimus lumborum*	−
**Abdomen**		
	*M. obliquus externus abdominis*	−
**Hindlimb**		
	*M. tensor fasciae latae*	−
	*M. biceps femoris*	**+**
	*M. semimenbranosus*	−
	*M. gracilis*	−
	*M. semitendinosus*	**+**
	*M. peroneus*	**+**

### Lymphoid tissue and kidney of BASE-affected cattle do not contain infectivity

Finally, we investigated the presence of infectivity in the lymphoreticular and urinary system of experimentally and naturally BASE-affected cattle by inoculation of groups of Tgbov XV mice with 10% homogenates of spleen, cervical lymph node and kidney (Table 1). None of the mice developed clinical signs of neurological dysfunction and were culled close to their predicted life span (850 days after challenge). Post-mortem examination did not reveal any neuropathological change or PrP^res^ accumulation by immunohistochemistry or Western blot analysis following PTA precipitation.

## Discussion

This is the first report on the occurrence and distribution of infectivity in peripheral tissues of BASE-affected cattle. We found that different skeletal muscles (i.e., *M. longissimus dorsi*, *M. intercostalis* and *M. gluteus*) from experimental and natural BASE carried infectivity, whereas spleen, cervical lymph node and kidney did not, as highly susceptible Tgbov XV mice did not develop disease up to 850 days after challenge. Noteworthy, all BASE muscles used for inoculation transmitted disease, although the attack rate differed between experimental and natural cases. This difference is likely related to different prion titers in the two conditions, possibly due to different stages of disease. In fact, the experimentally infected cattle was sacrificed at the terminal stage when clinical signs were severe [8], while the cattle with natural BASE was identified through active surveillance at a pre-symptomatic stage.

Infectivity in skeletal muscles has been detected in various natural and experimental prion diseases, including sheep affected by classical and Nor/98 atypical scrapie [17], deer with chronic wasting disease [18] and rodent models of scrapie [19]. A large pathogenesis study on BSE-infected cattle in the UK failed to detect infectivity in muscle tissue at any time during the course of the disease by inoculation of inbred mice [20]. However, in a subsequent study with Tgbov XV mice, *M. semitendinosus* from a field case of clinically symptomatic BSE transmitted disease to one out of ten inoculated rodents. This low amount of infectivity was tentatively related to the terminal nerve fibres [21].

A major clinical feature of cattle experimentally infected with BASE is amyotrophy, as a result of motor neuron disease [8]. Although we were unable to detect PrP^Sc^ in the peripheral nerves of infected cattle and follow the kinetics of PrP spreading through the neural pathway, a study of intra-species transmission of a Japanese case of BASE showed that PrP^res^ was first detectable by immunoblot in the nerve roots and subsequently in the peripheral nerves [22]. PrP^res^ deposition in skeletal muscles has been found in a variety of prion diseases, including experimental scrapie in hamsters [23], natural scrapie in sheep [24], and mouse and primate models of BSE and CJD [25]–[27]. These reports agree that PrP^res^ in muscle tissue is associated with terminal nerve endings.

In our study, immunohistochemistry showed the presence of amorphous or granular, small PrP deposits in different skeletal muscles from both experimental and natural BASE-affected cattle. PrP aggregates were found in isolated muscle fibers with a scattered distribution which was at variance with the topology of PrP^res^ reported in the previous studies [23], [24], [26], [27]. Noteworthy, identical results were obtained using two different fixatives and immunostaining protocols. Since PrP^C^ is expressed in bovine muscles and skeletal muscles are intrinsically capable of propagating prions [19], [28], we argue that PrP^Sc^ deposition in BASE muscles might be the result of a primary PrP replication through a neural-independent pathway. This possibility has been previously considered in a patient affected by sporadic CJD and inclusion body myositis where PrP^C^ to PrP^Sc^ conversion occurred in skeletal muscle [29].

The limited number and irregular distribution of PrP positive fibers within a muscle sample accounts for the absence of a detectable PrP^res^ signal on Western blot. The inhomogeneous distribution of PrP^res^ and infectivity among different muscles of the same animal and within the same muscle has been previously observed in mice and in primates infected with different prion strains. These findings have been tentatively related to biochemical differences in skeletal muscles of different body regions and/or number of nerve endings [19], [25], [26].

In the present study, Tgbov XV mice challenged with BASE muscles reproduced the monoglycosylated-dominant PrP^res^ type of BASE as well as a histopathological lesion profile and pattern of PrP^res^ deposition that matched those observed in mice challenged with BASE brain. These findings indicate that, in Tgbov XV mice, muscle and brain tissues maintain the same biological properties of the BASE strain.

Although serial transmission studies in Tgbov XV mice showed that the BASE prion is capable to replicate in the spleen converting, in part, into a BSE-like strain (personal observation), we did not detect infectivity in lymph node and spleen from cattle with experimental and natural BASE. This observation opposes the possibility that the BASE agent might re-circulate, potentially as BSE-like strain, in different hosts. Kidney is an edible organ and infectivity found in urine is kidney-associated [30]. Lack of transmission from kidney of natural and experimental BASE is an important issue, since infectivity in kidney has been demonstrated by bioassay in human prion diseases [31], and PrP^Sc^ has been observed in kidney of scrapie infected sheep and CWD affected deer [32]–[34].

The present data offer novel information on the tropism of the BASE agent and highlight relevant public health issues. While the transmission barrier for classical BSE is high in most species, BASE prions are readily transmissible to a variety of mammals including non-human primates [11]–[13], [35]. Accordingly, the possibility of spreading of BASE prions through skeletal muscle to other species should be taken into account and evaluated in risk analysis studies.

## Materials and Methods

### Ethics statement

This study was carried out in strict accordance with guidelines of the Italian Ministry of Health. The use and care of mice followed the EU directive 86/609/EEC (Council Directive of 24 November 1986 on the approximation of laws, regulations and administrative provisions of the Member States regarding the protection of animals used for experimental and other scientific purposes) and the Italian Legislative Decree 116/92 (Gazzetta Ufficiale della Repubblica Italiana 10, 18 February 1992). The study, including its Ethics aspects, was approved by the Italian Ministry of Health (Permit Number: NP-01-06). All surgery was performed under sevoflorane anesthesia, and all efforts were made to minimize suffering.

### Animal tissues sampling

Different tissues were collected from an Alpine brown cow (#995) that was experimentally infected by intracerebral inoculation of 10% brain homogenate from a cow with natural BASE and culled at the terminal stage of disease [8]. In addition, a variety of tissues was obtained from the third (#12966/07) and fourth (#126752/09) cases of natural BASE identified in Italy in 2007 and 2009 (Tables 1, 2, 3). These were an asymptomatic 14-year-old Piemontese cow and an asymptomatic 13-year-old Holstein-Friesian cow identified through active surveillance using a rapid screening test (PRIONICS-CHECK PrioSTRIP, Prionics, Zurich, Switzerland); in both cases the diagnosis was confirmed by histology, PrP immunohistochemistry and Western blot analysis.

Each tissue sample was cut in two halves, one of which was fixed either in Carnoy solution or 10% formaldehyde whereas the other one was frozen at −80°C. Samples of the same tissues were collected from three regularly slaughtered, normal cattle and used as negative controls.

### Transmission studies

Groups of Tgbov XV mice [15] were injected by a combination of i.c. (20 µl) and i.p. (100 µl) routes with 10% homogenate of occipital cortex and extracts prepared from different peripheral tissues from experimental BASE (#995) including spleen, kidney, cervical lymph node and *longissimus dorsi* muscle, and from the natural BASE (#12966/07) including spleen, kidney, and *intercostalis* and *gluteus* muscles. Five hundred milligrams of each sample were homogenized manually using a teflon-on-glass (Potter-Elvehjem) homogenizer (VWR International, Radnor, Pennsylvania, USA); the resulted suspension was clarified with low-speed centrifugation (1,000×g) for 1 min, using an Eppendorf 5418 centrifuge (Eppendorf s.r.l., Hamburg, Germany), and the pellet discarded. None of the peripheral tissues used for inoculation showed PrP^res^ by Western blot analysis without or with PTA enrichment.

All inocula were prepared under strict aseptic conditions in a microbiological containment level 3 laboratory, using disposable material for each inoculum to avoid cross-contamination. Groups of mice inoculated with BSE brain and normal cattle brain were included in the study as controls. Behavioral monitoring was carried out twice a week for detection of the onset of clinical symptoms and included spontaneous locomotor activity in the open field, reactivity to external stimuli and inverted screen test [36]–[38]. Clinically affected mice were sacrificed at the terminal stage of disease while all other mice were monitored for the entire predicted life span (up to 850 days) and then culled and subjected to autopsy.

### Histopathology and immunohistochemistry

At autopsy, the left lateral two-thirds of each mouse brain was fixed in Carnoy solution (ethanol∶chloroform∶acetic acid, 6∶3∶1) and serial 5-µm-thick sections from paraffin-embedded blocks were stained with hematoxylin-eosin (HE) and thioflavine S, or probed with the anti-PrP monoclonal antibody 6H4 (Prionics AG, Zürich, Switzerland, 1∶1000) and a polyclonal antibody to glial fibrillary acidic protein (Dakocytomation, Carpinteria, CA, USA, 1∶1000). Lesion profiles were established following the standard method described previously [39] using nine standard grey matter areas of the brains. Before PrP immunostaining, the sections were pretreated with proteinase K (Boehringer Mannheim, Ingelheim am Rhein, Germany) followed by guanidine isothiocyanate as reported previously [40]. Immunoreactions were revealed with the Envision System (Dakocytomation) for the polyclonal antibody or the Animal Research Kit Peroxidase (Dakocytomation) for the monoclonal antibody, using 3,3′-diaminobenzidine (Dakocytomation) as a chromogen.

Two different protocols were followed to immunostain the muscle samples, depending on the type of fixative used. Muscles from the Alpine brown experimental BASE cattle (#995) were fixed in 10% formalin and embedded in paraffin. Five-µm-thick sections were pre-treated following the protocol by Okada and co-workers [41] and probed with the anti-PrP monoclonal antibody F99/97.6.1 (VMRD Inc., Pullman,WA, USA, 1∶5000). The immunoreactions were visualized using an automated system Bond immunostainer (Vision BioSystems, Melbourne, Victoria, Australia) and a Fast Red/Alkaline phosphatase based reaction product.

Muscles from the Holstein-Friesian natural BASE cattle (#126752/09) were fixed in Carnoy. Five-µm-thick sections from paraffin-embedded blocks were pre-treated with proteinase K followed by guanidine isothiocyanate as described previously [40] and probed with the monoclonal antibody F99/97.6.1 (1∶1000). PrP^res^ immunoreactivity was visualized using a biotinylated anti-mouse antibody and the avidin-biotin peroxidase complex (Vector Laboratories, Burlingame, CA, USA, 1∶200), and 3,3′-diaminobenzidine (Dakocytomation) as chromogen; the sections were counterstained with Meyer's hematoxylin.

To verify the specificity of immunoreactions, the antibody F99/97.6.1 was pre-absorbed with a synthetic peptide homologous to residues 228–233 of bovine PrP before immunostaining. In order to maximize the possibility to detect PrP^res^ deposition in muscle fibres, three serial sections from experimental and natural BASE cattle were collected at 25 µm intervals. The two sections adjacent to the one selected for immunohistochemisty were stained with hematoxylin-eosin and X-34, an amyloid-binding, fluorescent derivative of Congo red [42].

### Western blot analysis

Frozen mouse brain and cattle muscle samples were homogenized in nine volumes of lysis buffer (100 mM sodium chloride, 10 mM EDTA, 0,5% Nonidet P-40, 0,5% sodium deoxycholate in 10 mM Tris-HCl, ph 7.4) and digested with 50 µg/ml of proteinase K (Boehringer Mannheim) for 1 h at 37°C. Digestion was blocked by addition of phenylmethylsulfonylfluoride. Samples were resolved using 12,5% polyacrylammide gels and then transferred onto PVDF membrane (Immobilon P, Millipore, Bedford, MA). Membranes were blocked with 5% non-fat dry milk in 10 mM Tris, 150 mM sodium chloride, 0.1% Tween-20, ph 7.5, for 1 hour and incubated overnight at 4°C with the monoclonal antibody 6H4 (Prionics, 1∶5000). Blots were developed using the Amersham enhanced chemiluminescence system (GE-Healthcare Ltd., St. Giles, UK) and visualized on an autoradiographic film. To enhance PrP^res^ detection, samples that were negative on a standard immunoblot analysis, were subjected to PTA precipitation as previously described [16] and analyzed by Western blot.

### Statistical analysis

Statistical analysis was performed using the GraphPad-Prism software. Kaplan-Meier survival curves were plotted, and differences in survival between groups of mice inoculated with different tissues were compared using the log-rank test.
